# The relationship between non-permanent migration and non-communicable chronic disease outcomes for cancer, heart disease and diabetes – a systematic review

**DOI:** 10.1186/s12889-019-6646-z

**Published:** 2019-04-15

**Authors:** Karen H. Wang, Zoé M. Hendrickson, Cynthia A. Brandt, Marcella Nunez-Smith

**Affiliations:** 1 Section of General Internal Medicine, Yale School of Medicine, PO Box 208093, New Haven, CT 06520 USA; 2 Equity Research and Innovation Center, Yale School of Medicine, PO Box 208093, New Haven, CT 06520 USA; 3 Veterans Affairs Connecticut Healthcare System, West Haven, CT USA; 4 Health, Behavior and Society Department, John Hopkins Bloomberg School of Public Health, Baltimore, MD USA; 5 Center for Medical Informatics, Yale School of Medicine, New Haven, CT USA

**Keywords:** Non-permanent migrants/migration, Geographic mobility/migration, Non-communicable chronic disease

## Abstract

**Background:**

The relationship between migration and health has primarily focused on permanent migrants, although non-permanent migrants comprise a large proportion of global migrants. Non-permanent migrants may have distinct needs that affect their health outcomes. This systematic review 1) examined the evidence concerning whether non-permanent migrants have different health outcomes than other population groups for non-communicable chronic diseases (NCDs) and 2) sought to describe how non-permanent migration is defined and measured.

**Methods:**

For this systematic review, we developed a comprehensive search string for terms about non-permanent migration and disease and screening rates for three NCDs (cancer, heart disease, and diabetes) and searched thirteen electronic databases using the search string. Authors reviewed and evaluated articles for full-text review; hand-searched specific journals and grey literature; and scanned reference lists of relevant studies. Authors extracted and assessed data based on standard reporting for epidemiologic studies.

**Results:**

We identified twelve peer-reviewed articles that examined NCD outcomes for non-permanent migrants as compared to other populations. Some studies showed worse or no significant differences in the NCD outcomes for non-permanent migrants compared to other groups. The articles reflected substantial diversity that exists among non-permanent migrants, which ranged from economic migrants to nomadic populations.

**Conclusion:**

Non-permanent migrants varied in their NCD outcomes as compared to other groups. Our included studies were heterogenous in their study designs and their definitions and measurement of non-permanent migration, which limited the ability to make conclusive statements about the health of the populations as compared to other populations. More standardization is needed in research to better understand the diversity in these populations and quantify differences in risk factors and disease rates between non-permanent migrants and other groups.

## Background

Rapid economic development has resulted in sustained global migration in the past several decades. The United Nations Development Program estimates that nearly 800 million migrants have moved permanently to either another country or another location within their own country’s borders for the purpose of resettlement [1, 2]. The impact of migration on economics and development has been a long-standing focus of the global community [3, 4]. The spread of communicable diseases from influenza to HIV continues to highlight the public health implications of migrating communities on health and health systems [5]. In the context of an increasing global burden of non-communicable chronic diseases (NCDs) [6], migrating communities will add an additional complexity to the role of health systems in improving population health.

The vast majority of NCD research among migrants has concentrated on identifying and addressing health needs and improving the healthcare of “permanent” migrants, i.e. immigrants (those who have resettled in another country) or rural-to-urban migrants (who have resettled within their country’s border) [7, 8]. Among NCDs, research has primarily focused on cancer, cardiovascular diseases, including diabetes, as they are the leading contributors to death worldwide [9–12]. With this large body of literature, some evidence suggests immigrants have better health outcomes than local populations, known as the ‘healthy migrant effect’ [13]. The suggestion is that there may be a selection effect, wherein healthier migrants are more likely to immigrate to another location, and thereby have better health outcomes than the local, non-migrant population [14, 15]. Studies have demonstrated, for example, lower cardiovascular disease and risk factors among immigrants as compared to locally born populations [13, 14, 16]. In contrast, other evidence suggests that immigrants continue to have poorer outcomes compared to local populations [7, 8, 17–19], such as higher rates of diabetes among immigrants in Canada or among Afro-Caribbean immigrants as compared to non-immigrant white populations in different settings globally [20–22]. Similarly, research on rural-to-urban migrants has documented this same equivocal trend in NCD rates of disease across differing rural populations [23–25]. These differences in the burden of NCDs and their complications between permanent migrant populations and non-migrant populations are important to understand to guide necessary prevention and management strategies [26, 27].

Another large body of research focuses on identifying and reducing NCD disparities in healthcare, specifically NCD screening disparities between permanent migrants and other populations [28]. A study in Canada, for example, demonstrated varying rates of diabetes screening in immigrants, with some sub-populations of immigrants having lower screening rates than the non-immigrant population [29]. Other studies of cancer disparities have demonstrated differential rates of cancer diagnosis in immigrant populations as compared to non-immigrant populations. Furthermore, many studies have demonstrated lower rates of cancer screening among immigrant populations as compared to other population groups [30, 31].

As migration is a dynamic process not often captured by literature on permanent migrants, there is a need to better understand the sub-populations often aggregated into the category of “migrants” [32]. Some researchers have explored migration-related attributes beyond permanent migration and their relationship to NCD outcomes to shed light on health differences between migrant populations and other populations [2, 33–35]. These attributes include, but are not limited to, length of time outside of place of origin; distance from place of origin; or the unit of migration (e.g., an individual or an entire family) [36]. Type of migration, other than “permanent” migration, is also a migration-related attribute that can influence health outcomes [36–45]. The “non-permanent” migrant populations (such as those who are temporary, circular, return, or double-leap/secondary migrants) are important to examine because: 1) with globalization, the volume of “non-permanent” migrant population is likely to grow; and 2) they likely represent a heterogeneous population who are at risk for NCDs [32, 38, 46].

We hypothesized that non-permanent migrants would have higher rates of disease and lower rates of chronic disease screening, as the dynamic livelihoods of non-permanent migrants may be a signal of greater vulnerability as compared to other migrant populations, based on World Health Organization conceptual model for the influence of social and structural determinants of health on the health of individuals [47]. Different than permanent migrants, the geographic mobility of non-permanent migrants may be a manifestation of the influence of contextual factors in different localities (such as discrimination, legal status, economic opportunity, available housing, etc.) and may independently affect health and healthcare outcomes, positively or negatively. The identification of greater rates of disease in non-permanent migrant populations as compared to other populations is essential to inform strategies to prevent and manage NCDs for these populations across geographic contexts. Moreover, identification of lower rates of screening in non-permanent migrant populations would necessitate a reassessment of the delivery of healthcare services to these populations.

As a step to advancing scientific understanding of these populations and their healthcare needs, we conducted a systematic review focused on the health of non-permanent migrants. Specifically, we examined the literature to compare non-permanent migrants to other populations for the NCDs, specifically cancer, heart disease, and diabetes, which are the main NCD contributors to mortality globally [9–12]. We were interested in rates of disease and screening as initial steps to understating health and healthcare needs. Since we anticipated variation in how non-permanent migrant populations were described in the literature, we also sought to describe how non-permanent migration was defined and measured.

## Methods

### Search string

We developed a comprehensive search string to identify articles measuring the relationship between non-permanent migration and disease and screening rates for three non-communicable diseases: cancer, heart disease, and diabetes (Table 1). Search terms for non-permanent migration included variations of terms that describe the process of migration, attributes related to migration, populations who migrate, and the main non-communicable diseases of interest.

**Table 1:** Examples of keywords and Boolean operators used in electronic database searches

Or ↓	<Migration>	And →	<Cancer>	Or →	<Heart Disease>	Or →	<Diabetes>
	*Subject headings*						
	Human migration^a^		Neoplasms^a^		Heart Diseases^a^		Diabetes Mellitus^a^
	Emigrants and immigrants^a^						
	Transients and migrants^a^						
	Residential Mobility^b^						
	Migrant/foreign worker^c^						
	Medical Tourism/Travel medicine^a^						
	*Text words (concepts to cover different types of migration by attributes)*						
	Geography		Tumor		Cardiovascular disease		Diabet*
	• Borders/Cross-border • Bi/transnational • Rural/urban • Regional						
	Patterns/Frequency		Cancer		Ischemic heart disease		
	• Return • Temporary • Regular • Circular						
	Populations		Carcino*/Onco*		Myocardial Infarction		
	• Mobile • Floating • Seasonal • Worker/farmworker						

^a^Medline subject heading. Similar subject headings were found and used in other databases where available

^b^CINAHL subject heading. Similar subject headings were found and used in other databases where available

^c^Embase subject heading. Similar subject headings were found and used in other databases where available

We identified relevant studies by searching thirteen electronic databases using the developed search string, hand-searching specific journals and grey literature, and scanning reference lists of relevant studies. We adapted the developed search string for the following databases: Medline, Embase, Global Health, PsychInfo, EBSCO, CINAHL, Africa-Wide NiPad, Sociological Abstracts, Social Services Abstracts, EconLit, IBSS, Latin American and Caribbean Health Sciences Literature Database, and Social Science Citation Index (See supplemental appendix for detailed search string for each database). We hand-searched migration-specific journals that were not indexed in the above databases, including *Asian and Pacific Migration Journal*, *International Migration Review, Journal of Borderland Studies, and Journal of Ethnic and Migration Studies*. Articles that cited relevant articles were also hand-searched according to inclusion and exclusion criteria. Lastly, we developed a comprehensive list of relevant organizations’ websites and searched for grey literature according to inclusion and exclusion criteria (available upon request).

### Inclusion and exclusion criteria

We retrieved articles published or released between January 1, 2003 and June 19, 2015, with titles and abstracts available in English. We reviewed all full texts available in English, Spanish, French, and Chinese. We included full-text articles with experimental and observational study designs. Full text articles eligible for final review included: 1) adults aged 18 years and older; 2) non-permanent migrants; 3) comparisons between a non-permanent migrant population and another population; 4) at least one non-communicable disease of interest, specifically diabetes, cardiovascular disease, or cancer; and 5) at least one measure of NCD outcome, i.e. disease or screening rates of three NCDs (prevalence or incidence measures).

For this study, to distinguish from a definition of permanent immigration used in peer-reviewed literature describing a lasting change in usual residence, we defined a non-permanent migrant as someone who had more than one movement across a border, suggesting a non-lasting residence [48, 49]. The term migrant is “understood to cover all cases where the decision to migrate was taken freely by the individual concerned for reasons of ‘personal convenience’ and without intervention of an external compelling factor, moving to another country or region to better their material or social conditions and improve the prospect for themselves or their family” [50]. Based on this definition, we excluded studies on refugees and asylum seekers. We further excluded undocumented migrants due to the unique barriers to care and health outcomes often experienced by these populations.

### Study selection

Once articles were pulled, we removed duplicate documents from the different databases. Two authors in an un-blinded systematic process evaluated the eligibility of abstracts for full-text review. They independently evaluated a sample of 100 abstracts to ensure the inter-rater reliability based on the inclusion and exclusion criteria (91% agreement). In the event of arbitration, the full text was pulled for further review.

Both reviewers extracted data independently from the final list of articles based on a pre-defined extraction form. For assessment, we adapted tools from the STROBE reporting for epidemiologic studies and Downs and Black [8, 51, 52]. We assessed each article for 1) completeness, based on the 22-item checklist from the STROBE, and 2) quality, based on an 11-item checklist from the Downs and Black tool [51]. The 22-item checklist assessed the information present in the title, abstract, background, methods, results, discussion, and funding reported by the authors. The 11-item checklist assessed quality by measuring risk of bias in external and internal validity [51]. The two authors scored the final set of articles independently and then averaged the scores.

## Results

**Fig. 1:**
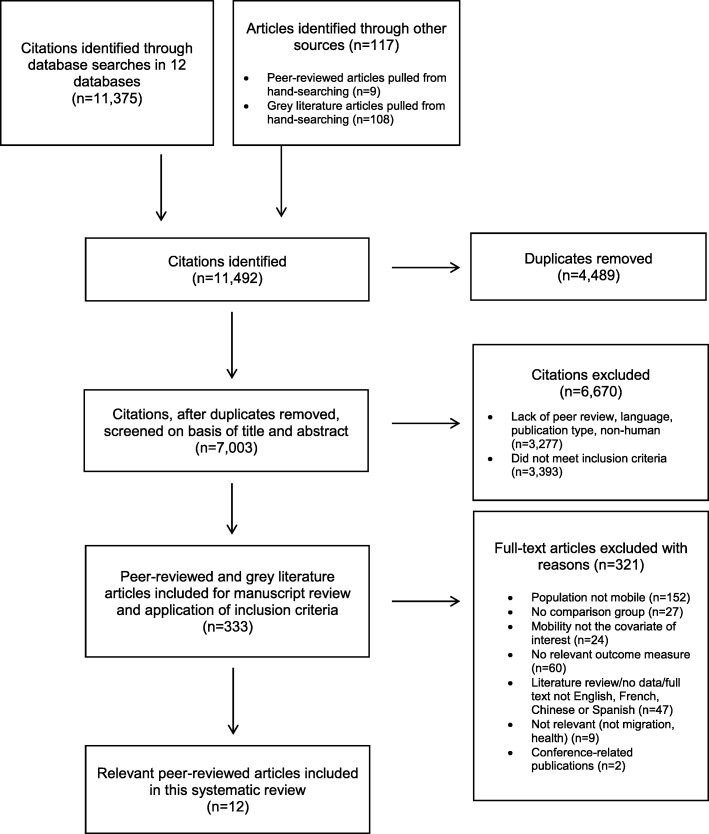
Flow Diagram

The search of databases provided a total of 11,492 citations (Fig. 1). Our review of grey literature and non-indexed journals resulted in zero papers for inclusion. Twelve articles met our inclusion criteria [40, 53–63]. The number of abstracts and full texts reviewed at each stage are shown in Fig. 1.

### Synthesis of results

#### General description of included studies

The 12 studies reflected a range of non-permanent migrant groups examined and study designs in the peer-reviewed literature (Table 2) [40, 53–63]. The non-permanent migrant groups examined included: three studies on migrant farmworkers in in the United States, who traveled across regions with the growing seasons [54–56]; one study on female non-local sex workers who had temporary visas to live in Hong Kong, China [63]; four studies on nomadic populations (the Traveller population in Ireland and the United Kingdom and Mongolian pastoralists) [58–61]; three studies of return migrants in Mexico, who had moved from Mexico the United States and at some point returned back to Mexico [40, 53, 62], and one study on repatriates in Norway [57]. Eleven studies had a cross-sectional design [40, 53–56, 58–63], and one study was a retrospective cohort study [57].

**Table 2:** Peer-reviewed articles on non-permanent migration included in the analysis (*N* = 12)

Reference (* signifies reference # in manuscript)	Study design	Migrant population	Geographic border	Measurement of non-permanent migration	Outcome of interest	Relevant results (* = *p* < 0.05, ** = *p* < 0.01 *** = *p* < 0.001; ^=*p* > 0.05)	Summary	Reporting score (max = 22)	Quality score (max = 11)
Aguila et al. 2013 ([53]*) doi:10.1177/0898264312468155 MESH: Emigrants and Immigrants	Cross-sectional study using data from the 2003 Mexican Health and Aging Study (MHAS) and the 2004 Health and Retirement Survey (HRS) to compare diabetes outcomes between migrant groups.	MHAS: Return migrants: 1482 Mexican-born people who migrated to US and returned to Mexico Non-migrants: 11,054 people HRS: Immigrants to U.S: 505 people	National: Migration between United States and Mexico	Migrant status: Return migration is at least 2 moves (from Mexico to the US and from US back to Mexico)	Diabetes (self-report)	Men: return migrants compared to immigrants to the US (AOR 0.59, CI 0.26–1.33); non-migrants compared to Mexican immigrants (AOR 0.75, CI 0.31–1.80) Women: return migrants compared to immigrants to the US (AOR 0.87, CI 0.40–1.85); non-migrants compared to Mexican immigrants (AOR 0.85, CI 0.42–1.71)	Return migrants and non-migrants in Mexico were as likely to report diabetes as immigrants to the United States.	21	8
Castaneda et al. 2012 ([55]*) doi: 10.1177/2150131911422913 Keyword: farmworkers	Cross-sectional study using survey data from 2002 to 2004 to compare cancer screening rates between migrant and seasonal farmworkers.	173 migrant and seasonal farmworkers Women age > 40	Regional (Intra- national): Migration between Michigan, US, and other regions in the US	Migrant status: U.S. Department of Labor’s definition of migrant farmworker status (travels across regions following growing seasons). Other group: seasonal farmworker status (people living in one area who are residents and work on farms during growing/harvest season)	Cancer: Breast/cervical (self-report and chart review)	1) Ever had a mammogram: seasonal farmworkers (100%) > migrant farmworkers (70.6%).** 2) Pap test (ever or within past year): No difference between groups.^	Migrant farmworkers were less likely to have received a mammogram as compared to permanent seasonal farmworkers.	15.5	4.5
Castaneda et al. 2015 ([54]*) doi:10.1080/1059924X.2015.1010060 Keywords: migrant, farmworkers	Cross-sectional study using 2002–2004 data from Oceana Farmworker Health Survey to examine CVD risk factors comparing migrant and seasonal farmworkers in Michigan.	300 farmworkers self-identifying as a migrant (non-permament) or seasonal farmworker (permanent), were recruited through mailings to residences at migrant camps as well as residences outside the camps.	Regional: See Castaneda et al. 2012	See Castaneda et al. 2012	Diabetes: Diagnosis as part of a questionnaire (self-report)	Migrant (7.0%) < Seasonal farmworkers (11.5%) ^	Non-permanent/ migrant and permanent /seasonal farmworkers did not have a statistical difference in their prevalence of diabetes.	21.5	5.5
Knoff et al. 2013 ([56]*) doi:10.1177/21501319-13476303MESH: Transients and Migrants	Cross-sectional study to compare cervical cancer screening for Hispanic migrant and seasonal farmworkers in Michigan.	309 eligible patients living in two communities in Michigan (May 2011–August 2011) comparing migrant farmworkers (non-permanent) to seasonal farmworkers (permanent)	Regional (Intra-national): Migration between Michigan, US and other regions in the US	Migrant status: migrant farmworkers defined as those who reported that in the last year, they had traveled out of Michigan to work Other group: all others were defined as seasonal farmworkers	Cancer: Cervical Measured as 1) ever Pap test and 2) a recent Pap test (self-report).	Migrant and seasonal farmworkers did not have significantly different rates of either 1) ever having a Pap test or 2) having a recent Pap test (88% vs. 87.4).^	There was no difference in rates of cervical cancer screening between non-permanent/migrant farmworkers and permanent/ seasonal farmworker groups.	20	6
Kristensen & Bjerkedal 2010 ([57]*)doi:10.1007/s10654-009-9417-9MESH: Emigration and Immigration	Retrospective cohort study of Norwegians between 1967 and 1976 with follow-up January 1992 to December 2004 using multiple national registries (birth, cancer, and cause of death registry, fd-trygd) to assess mortality and incidence of cancer among different groups of migrants.	626,928 individuals separated into three groups based on available data: 1) non-emigration, 2) emigration to another country, and 3) repatriation	National: Migration between Norway and other international destinations	Migrant status Migration history included measure of person-time after repatriation Other groups: person-time 1) before emigration; 2) during emigration	Cancer: First incident cancer	Cancer (per 100,000 person-years): 66.9 after return/repatriation vs. 13.2 during emigration vs 57.0 before emigration). Adjusted rate ratios comparing emigrants to non-emigrants (0.19, CI 0.13–0.30) and return migrants to non-emigrants 0.80 (0.58–1.11).	There was no difference in cancer incidence between non-permanent/return migrants and non-migrants; there was a difference in cancer incidence between permanent migrants and non-emigrants	21	10
McGorrian et al. 2011 ([58]*) doi:10.1177/1741826711428059MESH: Transients and Migrants	Cross-sectional study of the All-Ireland Traveller Health Study (AITHS) to compare cardiovascular disease (CVD) and CVD risk factors in the Traveller population to the general population in Ireland.	1878 adult Travellers compared to 3445 participants from the Irish population survey with lower socioeconomic status. The Traveller population to the general population	Regional (intra-national): Migration within regions of Ireland	Migrant status Traveller: defined as an indigenous minority population with a nomadic tradition Self-identification as a Traveller was the measure	Diabetes (self-report); CHD (self-report)	Diabetes: Traveller rate (10.9) > general population (4.6%)***CHD: Age-adjusted Traveller rate (12.7%) > general population (12.5). ^	The non-permanent migrant Traveller population has a higher prevalence of diabetes than the general population.	21.5	7
Mocellin & Foggin 2008 ([59]*) doi:10.1016/j.healthplace.2007.06.005MESH: Transients and Migrants	Cross-sectional study using data from 1992 to 1994 on the relationship between geographic mobility of semi-nomadic pastoralists and health outcomes.	615 households and 3167 individual nomadic pastoralists from three diverse provinces in Mongolia.	Regional (intra-national): Migration within regions of Mongolia	Migrant status Semi-nomadic pastoralism: “implies extensive pastoralism based on periodic changes of pastures during the course of the entire or the greater part of the year” Included several migration/geographic mobility attributes: 1. Distance traveled 2. Temporal character of the migration	CHD: symptoms related to heart disease (self-report)	Participants reporting geographic mobility had a significantly greater risk of symptoms related to CHD than those without mobility (AOR: 1.65).*** Those reporting frequent movements were 2.96 times more likely to have symptoms related to CHD than those moving less frequently.*	Higher geographic mobility was associated with greater CHD symptoms.	19	6
Parry et al. 2007 ([60]*) doi:10.1136/jech.2006.045997 MESH: Transients and Migrants	Cross-sectional study of Roma and Travellers’ health outcomes to determine whether health inequities are explained by socioeconomic disadvantage or ethnic minority group membership.	260 Roma and Travellers in the United Kingdom (non-permanent migrant) compared to 260 individuals of low socio-economic status, rural and urban status, Pakistani origin; or African Caribbean origin.	Regional (intra-national): Migration within UK	Migrant status Identification as Roma or Traveller vs. not Also measured whether Travellers travel all year, in the summer, or rarely travel	CHD: Symptoms of chest pain, possible angina (self-report based on Rose Angina Scale); Diabetes (self-report)Cancer: (self-report)	CHD: Chest pain/discomfort: Roma and Travellers 34% vs. comparison group 22%.** Possible angina symptoms: Roma and Travellers 30% vs matched comparison group 20%.** Heart disease (including angina): Roma and Travellers 8% vs. matched comparison group 4%.*No significant differences reported for diabetes or cancer.	The non-permament Roma and Traveller populations have higher rates of angina symptoms as compared to individuals of low socio-economic status, rural and urban status, Pakistani origin; or African Caribbean origin.	19.5	4
Peters et al. 2009 ([61]*) doi:10.1080/13557850802699130 Found from review of references	Cross-sectional study to compare Roma and Travellers’ health outcomes with other ethnic minorities and low-income populations.	See Parry et al. 2007	See Parry et al. 2007	See Parry et al. 2007	CHD: Parry et al. 2007	CHD: African Caribbean 32% > Roma and Travellers 30% > Whites 18% > Pakistani Muslim 12%.** Multivariate analyses found that Pakistani group had reduced odds compared to Roma and Travellers.*	The non-permanent migrants Roma and Traveller populations have worse outcomes as compared to some but not all comparison groups	19	5
Riosmena et al. 2013 ([62]*) doi:10.1007/s13524-012-0178-9MESH: Emigration and Immigration	Cross-sectional study using two nationally representative datasets: The National Health Interview Survey (NHIS, 1997–2007) in the US; the Mexican Health and Aging Study (MHAS, 2001) in Mexico to compare the prevalence of diabetes between different migrant populations.	MHAS: 5138 Mexican men, 382 with history of living in Mexico with United States. NHIS: 39,985 White men, 1729 United States-born Mexican-Americans, and 1328 Mexican immigrants. Compare four groups: US-born Mexican-American men, non-Hispanic white men and Mexican-born men (non-migrant and return migrant).	National: Migration between US and Mexico	Migrant status: Return migration (not specifically defined) implied as those who have emigrated and then returned to the original sending country of origin Duration of stay: in years in receiving country; for return migrants (during since return to sending country)	Diabetes: Ever diagnosed (self-report)	No difference in diabetes between groups: Analyses adjusted for age, education, and time since return (for return migrants). Longer US experience had greater odds of diabetes compared to shorter US experience.	No difference in prevalence of diabetes between the three groups (non-migrants, return migration, and immigrants).	20.5	8
Ullmann et al. 2011 ([40]*) doi:10.1016/j.socscimed.2011.05.037MESH: Transients and Migrants	Cross-sectional study using data from the Mexican Migration Project in Mexico (2007–2009) to compare health status for returned migrants vs. non-migrants.	2121 men (heads of household) from 14 locations in Mexico.	National: Migration between United States and Mexico	Migrant status Return migrants): Defined as people with migration experience to the US but are in Mexico at the time of the survey Migration trips Number of trips a head of household took to the US(A trip was defined as a visit to the US for work, job search, or “stable residency.” Short visits were not included) Duration of stay Measured as number of months living in the US	Diabetes (self-report); CHD: Heart attacks (self-report)	Return migrants were significantly more likely than non-migrants to have experienced a heart attack or heart disease (6.9% vs. 3.6%).* They were more likely to have diabetes/high blood sugar (12.0% vs. 9.9%) but this difference was not significant.^In multivariate analyses adjusting individuals with migration experience had 2 times greater odds of having had a heart attack/heart disease than those without.**	Return migrants were more likely to report heart disease.	22	9
Wong et al. 2008 ([63]*) Doi: 10.1111/j.1525-1438.2007.00970.x keyword: migrant	Cross-sectional study (January 2004–December 2005) on cervical cancer screening among female sex workers (FSWs) and migrant workers.	245 FSWs screened at an outreach clinic in Hong Kong.	Regional (intra-national): Migration between Hong Kong and China	Migrant status Non-local FSWs were defined as those with a temporary “visitor’s visa” Other group: New immigrants were defined as “local FSWs”	Cervical Cancer:(based on pap smear results)	Compared to local FSWs, non-local FSWs were less likely to receive a Pap smear (19.2% vs. 42.6%),* more likely to have abnormal Pap results,* and higher grade cervical change in multivariate analyses (AOR: 17.13).*	Temporary migrants were less likely to have a Pap smear, abnormal Pap results, and higher grade cervical changes.	17	5

### Outcomes of interest

#### Prevalence/incidence rates of NCD

Among the 12 studies comparing non-permanent migrant population to at least one other population, one article examined differences in prevalence rates of cancer, heart disease, and diabetes [60]; two articles, heart disease and diabetes [40, 58]; one, cancer incidence only [57], two articles, heart disease only [59, 61], and three articles, diabetes only [53, 54, 62],

Parry et al. examined three health outcomes of the non-permanent Traveller population as compared to age and sex-matched individuals (who were either ethnic minority, low socioeconomic or non-urban populations) [60]. In bivariate analyses, this study demonstrated higher heart disease in the non-permanent Traveller population as compared to the comparison group (prevalence: 8% vs 4%, *p* < 0.05), but it found no differences in rates of diabetes or cancer between the groups [60]. McGorrian et al. compared rates of heart disease and diabetes in another Traveller population to a general population and found no significant difference between self-reported rates of heart disease but did find a significant difference in diabetes rates (10.9% vs. 4.6%, *p* < 0.001) [58]. Ullmann et al. compared self-reported heart disease and diabetes between non-migrants in Mexico and non-permanent return migrants to/from Mexico and United States [40]. The non-permanent return migrants were significantly more likely to report heart disease than non-migrants (6.9% vs 3.6%, *p* < 0.05) [40].

The retrospective cohort study by Kristensen et al. examined cancer incidence among a nationally representative sample of residents in Norway across three groups (non-migrants, emigrants, and repatriates) and demonstrated no statistical difference in cancer incidence between non-migrants and non-permanent repatriates [57].

Studies by Aguila et al. and Riosmena et al. showed no difference in the odds of self-reported diabetes when comparing return migrants to two other population groups (non-migrants who remained in Mexico and immigrants in the United States from Mexico) [53, 62].

#### Screening rates of NCDs

Three studies measured breast and cervical cancer screening rates among non-permanent migrants compared to another population group [55, 56, 63]. Studies by Castenada et al. and Knoff et al. comparing non-permanent migrant farmworkers to seasonal farmworkers in the United States (i.e. as those who lived in one place and worked as farmworkers during the harvest season) demonstrated similar rates of cervical cancer screening between population groups [55, 56]. However, in the same Castenada et al. study, the screening mammography rates were significantly lower in the non-permanent migrant farmworkers as compared to the seasonal farmworkers (70.6% vs 100%, *p* < 0.01) [55]. Wong et al. compared non-permanent migrant female sex workers (i.e. those who had a temporary visa status) to other female sex workers in Hong Kong, China, demonstrating that the non-permanent migrant female sex workers were less likely to have had a pap smear and were more likely to have abnormal pap smear results than other female sex workers [63].

#### Definition and measurement of non-permanent migration

Definitions and measurement of non-permanent migration varied across studies. The majority of studies identified or assumed a person’s “migration status” based on movement across geographic borders (often international or intra-national borders) and traveling from one place to another location and living in this other location. For example, Kristensen et al. used terms related to movement across national borders, and groups of migrants were categorized as non-emigrants, emigrants, and repatriates [57]. Several studies, including Aguila et al., Riosmena et al., and Ullmann et al., used the term, “return migrants,” and Ullmann et al.’s study specifically defined “return or returned migrants” as “those in the sample that have previous migration experience to the U.S. but are in Mexico at the time of survey” [40, 53, 62]. In Castaneda et al. and Knoff et al., as described above, migrant status was based on a designation given by a governmental body, differentiating two different types of farmworkers based on the geographic mobility of one group (migrant farmworker) as compared to farmworkers “who live in one place” (seasonal farmworker) [55, 56]. Similarly, Wong et al. defined their migrant groups based on a legal definition, as determined by a person’s visa that determined length of stay [63]. A few studies expanded beyond identifying “migrant status” and measured distance traveled or discrete counts of crossing a regional or national border [57, 59, 62].

#### Study quality

Of the 12 studies, the completeness of reporting based on the STROBE 22-item checklist ranged from 15.5 to 22 [40, 53–63]. The quality of the studies, based on an 11-item checklist from the Downs and Black tool, ranged from 4.5 to 10, with lower scores most often due to poor generalizability to other populations because sampling was not representative of the entire population of interest. Moreover, 11 of the 12 included studies were cross-sectional design [40, 53–56, 58–63], among which only three studies clearly indicated how missing data might have affected study findings to address possible issues with confounding [56, 58, 62].

## Discussion

This systematic review of the literature found 12 studies that compared non-permanent migrants with another population to examine differences in NCD outcomes for cancer, heart disease, and diabetes. Our main finding was that NCD outcomes of non-permanent migrants were similar to or worse than other populations. However, these equivocal findings likely reflect other important conclusions of this review, such as the heterogeneity of 1) study designs, 2) non-permanent migrant groups by migration attributes and 3) spatial and temporal measurements of migration.

Some studies found significant differences in NCD prevalence between non-permanent migrant populations and other population groups. For example, of the four studies on heart disease, all documented higher rates of self-reported heart disease among the non-permanent migrants as compared to other groups [58–61]. In contrast, of the five studies on diabetes, only one study demonstrated that non-permanent migrants had higher prevalence than other non-migrant groups; the other studies showed no difference [40, 53, 54, 58, 62]. In light of the variation in study designs, measurements, and research contexts, it is challenging to make strong conclusions about differences between non-permanent migrant groups and other population groups. However, differences within non-permanent migrant groups and between these groups and others likely exist, as past literature on permanent migrants suggests multifactorial influences on disease risk including genetics, environmental exposures, and social-behavioral factors [35, 64]. For non-permanent migrants, we expect there may be factors affecting health risk and outcomes, complicated by the additional influence of having resided in different geographies, with varying numbers of movements, over different periods of time. Non-permanent migrants may also have social and family ties across geographies that influence health risks and outcomes [45, 65–68]. However, the strength of these ties may differ as a result of varying migration attributes, such as the migration unit (i.e. the individual or family), distance from one’s network, cultural identities and broader socioeconomic dynamics in countries of origin [69–71]. We infer that these contextual and social-level factors in these different geographies confer unique risks and opportunities that affect an individual’s health [38, 72–74]. Future work should investigate the influence of these factors on the health behaviors and practices of non-permanent migrants in different geographic locations.

The included studies represented diverse migrant populations in their motivations and their migration-related spatial and temporal attributes. For example, the studies represented a broad range of motivations that led to an individual’s non-permanent migrant status, including for labor reasons (e.g. farmworkers) and lifestyle (e.g. nomadic populations), that would affect outcomes [54–56, 58–61]. The geographic borders crossed were both regional and national and are important to identify and understand, as living in one context or another confers different opportunities and challenges for migrants. Moreover, if measured, the time period for the measurement of migration ranged from seasonal to discrete counts of a migration event [40, 57, 59, 60]. This issue has been discussed in migration literature, where the temporal attributes of migration such as frequency, duration, and timeframe are not consistently measured, thereby hindering opportunities for generalization and comparison in migration research [75].

A review of how the included were indexed within the bibliographic databases suggests a need for more granular and standardized vocabulary around non-permanent migration. Three of the 12 articles were retrieved from keyword searches with use of the terms “migrant” or “farmworker” [54, 55, 63], eight were retrieved based on their categorization into the broad medical subject headings like “Emigration and Immigration” and “Transients and Migrants” [40, 53, 56–59, 61, 62]. These findings are consistent with the broad International Office for Migration (IOM) definition of migration [2, 76]. A few other definitions exist that may serve to provide a framework for this population, but these are also limited. For example, per IOM, a short-term migrant is “a person who moves to a country other than that of his or her usual residence for a period of at least three months but less than a year, *except* in cases where the movement to that country is for purposes of recreation, holiday, visits to friends or relatives, business or medical treatment” [50, 77]. This definition excludes migrants who are regionally mobile within national geographic borders, those who may remain in one locality for more than one year, or those who have no usual residence. Refinement and standardization of the language used to describe non-permanent migration will enable further examination of the health behaviors and health outcomes of these populations.

There are several limitations to this systematic review. Our own a priori definition of “non-permanence” based on the number of moves across a border suggesting a non-lasting residence is a limited, but important, facet of non-permanence. Our premise was that these individuals were geographically mobile, and therefore may also be residentially mobile, to differentiate from the concept of “permanent” migrant whose definition is tied to having a usual residence. However, the concept of residence/residential mobility limits our understanding of non-permanence. ‘Residence’ is less central to the concept of non-permanent migration as compared with permanent migration, as non-permanent migrants may have no usual residence or no residence at all [49]. The definition utilized here demands further development and investigation to capture the diversity of different non-permanent migrant populations, to highlight the different motivations for migration, and to better understand the influence of contextual factors in different locations. However, these findings reflect the current limitations and challenges in this field of study, where there is wide variation in vocabulary, definitions, and measurement across disciplines and limited availability of data [35]. Article quality of included studies also varied and limited the ability to generalize or make inferences about the relationship between non-permanent migration as compared to other populations and NCD disease and screening rates. As prior research studies have noted, variations in terminologies, design, and quality challenges comparisons across studies and underscores the need of further systematization to establish standards of practice [35].

The limited number of studies on non-permanent migrants is not surprising. Some studies on non-permanent migrant populations (such as labor migrants, nomadic populations, and farmworkers) that were excluded from our final analysis did not include a comparison group [78–83]. Each migrant population has distinct individual and societal factors that influence health and health outcomes, which makes finding an appropriate comparison group challenging [35, 84]. The limited number of studies on non-permanent migrants from low- or middle-income countries likely reflects our inclusion criteria, such as requiring a comparison group or outcomes of interest [59, 81, 85, 86]. Though it is possible we did not capture all articles for inclusion into our review because of the diversity of terms for migration, to minimize this possibility, we created a comprehensive search string, reviewed articles in the references list of included articles, and reviewed relevant grey literature. Moreover, our analysis focused on cancer, heart disease, and diabetes, yet non-permanent migrants are affected by other non-communicable diseases, such as chronic obstructive lung diseases and obstetric diseases, that are influenced by social and structural determinants of health [87, 88]. Future work can investigate these populations in low- and middle-income countries and expand outcomes of interest.

More standardized measurements for the spatial and temporal attributes of non-permanent migration and explicit documentation of definitions are needed [57, 59, 62]. Consistent use of a multilevel framework for migration may inform standardization of variable selection and definitions used. In peer-reviewed literature on permanent migrants, several conceptual models have been used to define variables that influence individuals’ health outcomes [89–91]. These models could be adapted for research on non-permanent migrants to account for the multiple contexts in which non-permanent migrants have lived and how these contexts influence their health and health seeking behaviors. Researchers could also begin to consistently use the term “non-permanent” to describe these populations in distinct contrast to permanent migrants. With greater standardization, we can then incrementally build upon the limited knowledge about the different typologies of non-permanent migration, advance the science around migrating populations, and improve public health systems’ abilities to plan for and care for these populations.

## Conclusion

The confluence of non-permanent migration in the context of the growing burden of NCDs is an important area of investigation that requires greater examination. As research examining non-permanent migrants’ health grows, standardized vocabulary, definitions, and measurements of migration attributes are key mechanisms for organizing data to facilitate indexing and synthesis of results across multiple contexts. This work has implications not only for the health of migrant populations but also for the public health systems that care for these populations. Currently, health prevention, screening, and treatment protocols may vary across these locations, and health information about these non-permanent populations is not often accessible in different regional or international areas [92, 93]. Public health and healthcare systems may need to develop different strategies to minimize health risks of NCDs for non-permanent migrants across geographies.

## Abbreviations

IOM: International Organization for Migration
NCDs: non-communicable chronic diseases
